# The Relationship between Selenoprotein P and Glucose Metabolism in Experimental Studies

**DOI:** 10.3390/nu5061937

**Published:** 2013-05-29

**Authors:** Jinyuan Mao, Weiping Teng

**Affiliations:** Liaoning Provincial Key Laboratory of Endocrine Diseases, Department of Endocrinology and Metabolism, Institute of Endocrinology, The First Affiliated Hospital of China Medical University, No.155, North Nanjing Street, Shenyang 110001, China; E-Mail: jinyuanmao@hotmail.com

**Keywords:** selenium, selenoprotein P, glucose metabolism, insulin, gene expression

## Abstract

Selenium is an essential trace element in the diet of mammals which is important for many physiological functions. However, a number of epidemiological studies have suggested that high selenium status is a possible risk factor for the development of type 2 diabetes, although they cannot distinguish between cause and effect. Selenoprotein P (Sepp1) is central to selenium homeostasis and widely expressed in the organism. Here we review the interaction between Sepp1 and glucose metabolism with an emphasis on experimental evidence. In models with or without gene modification, glucose and insulin can regulate Sepp1 expression in the pancreas and liver, and *vice versa*. Especially in the liver, Sepp1 is regulated virtually like a gluconeogenic enzyme. Combining these data suggests that there could be a feedback regulation between hepatic Sepp1 and pancreatic insulin and that increasing circulating Sepp1 might be the result rather than the cause of abnormal glucose metabolism. Future studies specifically designed to overexpress Sepp1 are needed in order to provide a more robust link between Sepp1 and type 2 diabetes.

## 1. Introduction

Selenium is an essential trace element in the diet of mammals. While its importance for the brain [1], fertility [2], immune [3] and thyroid functions [4] and cancer prevention [5] is well established, the association between selenium and glucose metabolism is conflicting. Despite the expectation that selenium might prevent the development of diabetes owing to its antioxidant and insulin-mimetic properties [6,7,8], in recent years, some epidemiological studies, including cross-sectional studies and longitudinal studies, have shown that supranutritional selenium intake or high plasma selenium levels are a possible risk factor for the development of type 2 diabetes or metabolic syndrome [9,10,11,12]. However, there are also studies that support a decreased risk of type 2 diabetes at higher levels of Se intake [13,14,15]. In the prospective observational Epidemiology of Vascular Ageing (EVA) study, 1162 participants with complete data were analyzed and it was found that high plasma selenium correlated with a decreased risk of onset of hyperglycaemia during a 9-year follow-up period in elderly men (though not in women) [14]. However, most randomized controlled trials showed that selenium supplementation had no protective or adverse effect for type 2 diabetes [16,17,18], except the Nutritional Prevention of Cancer (NPC) Trial. In the NPC trial, compared to placebo, oral administration of selenium of 200 μg/day statistically significantly increased risk for type 2 diabetes but only in the population in the highest tertile of baseline plasma selenium (>121.6 μg/L) [19].

Selenoprotein P (Sepp1) is central to selenium homeostasis in the organism because it promotes the retention of selenium in the body and affects selenium distribution from the liver to extra-hepatic tissues [20]. Both Yang and Misu reported that circulating SEPP1 levels were associated with dysregulation of glucose metabolism in humans [21,22]. In the Korean study, serum SEPP1 levels were significantly higher in patients with type 2 diabetes (*n* = 40) or prediabetes (*n* = 40) than in those with normal glucose tolerance (*n* = 20) [21]. At the same time, overweight and obese subjects (BMI > 23 kg/m^2^) had increased SEPP1 levels compared with lean subjects (BMI < 23 kg/m^2^). The Japanese study by Misu *et al.* found that circulating SEPP1 levels were significantly correlated with fasting plasma glucose (*r* = 0.35, *p* = 0.037) in 36 patients with type 2 diabetes and negatively correlated with total and high-molecular weight adiponectin levels in these patients [22]. These studies hint that SEPP1 could be the link between selenium and type 2 diabetes. Here, we review the interaction between Sepp1 and glucose metabolism with the emphasis on experimental evidence which could underlie the cause-and-effect relationship of selenium and glucose metabolism. Moreover, with the development of animal models with genetic alterations, there are wider opportunities to elucidate the mechanisms under different conditions.

## 2. Expression of Sepp1 in Liver, Pancreas and Adipose Tissue

Selenium exerts biological functions mainly as the amino acid selenocysteine (Sec) in selenoproteins in mammals. SEPP1 is the only selenoprotein containing more than one Sec residue in its sequence. Up to 10 Sec residues are incorporated in the Sepp1 of rats, mice and humans; the *N*-terminal domain contains one Sec and the *C*-terminal domain contains multiple Sec (up to a maximum of nine) [23]. SEPP1 is a secreted glycoprotein that contains around 22%–65% of the plasma selenium content in humans, depending on individual selenium status [24,25].

Plasma SEPP1 concentration is the best marker of human selenium nutritional status [26]. Xia *et al.* reported that plasma GPx3 reached its maximum value when a supplement of 37 μg selenium, as selenomethionine, per day was given to Chinese men of low selenium status, while SEPP1 concentration continued to rise and did not reach a plateau even at the highest supplement dose, 61 μg/day [27]. However, once the nutritional requirement has been met, SEPP1 concentration does not reflect additional increases in selenium intake [27,28]. 

Sepp1 is widely expressed in many tissues of mice [29]. Liver has the greatest relative amount of Sepp1 expression of all mouse tissues [20] and is the main source of plasma Sepp1 [30,31], 90% of which originates in hepatocytes [20]. Selective deletion of Sepp1 in hepatocytes (from *Sepp1^c/c^/alb-cre*^+/−^ mice) impairs selenium supply to extra-hepatic tissues and worsens dietary selenium deficiency [20]. 

The pancreas is the only organ producing insulin; Sepp1 is present but mRNA levels are lower by nearly 50% than in rat liver [32]. Pancreatic expression in rodents is restricted to the islets and endocrine cell lines, is not produced in the exocrine pancreas tissue [32,33,34] and appears to be co-localized with both insulin and glucagon by immunofluorescence [32]; however, it is expressed more strongly in beta-cells than in alpha-cells [34]. Additionally, no secreted Sepp1 isoforms were detected in the supernatant from the rat insulinoma cell INS-1, which suggests that Sepp1 produced by pancreatic cells does not function as an external secretion as does liver. Nevertheless, we should realize that it is not always easily possible to extrapolate from rodent models to humans.

Adipose tissue has a central role in lipid and glucose metabolism and also expresses SEPP1. In a human study, SEPP1 gene expression was downregulated in subcutaneous adipose cells isolated from eight insulin-resistant patients, compared with eight insulin-sensitive subjects by microarray [35]. Likewise, in an animal study, Sepp1 gene was expressed at a significantly lower level in epididymal adipose tissue of either *ob/ob* mice or high-fat diet (HFD)-induced obese mice than in control mice on a regular chow diet. When HFD-induced obese mice were treated with the antidiabetic drug rosiglitazone, the protein level of Sepp1 was significantly elevated [36]. All these data indicate that the expression of Sepp1 gene in adipose tissue is negatively correlated with obesity and insulin resistance.

## 3. The Change of Sepp1 Expression with Glucose and Insulin in Models without Gene Modification

The concern about the relationship between selenium and glucose metabolism is that there may be adverse effects of selenium over-supplementation, given that selenium is commonly added to multivitamin/mineral preparations that are consumed by the general public in many Western countries. Supplementation with selenium has experimentally been shown to protect against certain cancers and reduce total cancer mortality [37,38], while high-dose sodium-selenite (1000 µg/day) reduces mortality rate in patients with severe sepsis or septic shock [39]. Campbell *et al.* found that sodium selenite and selenate at 30 nM stimulated the biosynthesis and secretion of insulin in Min6 insulinoma cells and isolated rat islets [40]. Steinbrenner and colleagues also observed that selenium compounds at 1 μM can significantly upregulate Sepp1 gene expression in INS-1 cells cultured under normoglycemic conditions (5 mM glucose) [32]. It could be speculated that selenium may stimulate insulin production mediated by increasing Sepp1 expression in pancreatic β cells. However, high glucose concentrations dose-dependently suppressed the selenium-induced elevation of Sepp1 mRNA level, by inhibiting Sepp1 promoter activity. On the other hand, it is observed that high glucose concentrations (11 or 22 mM), without treatment with extra selenium compounds, significantly down-regulated Sepp1 expression by more than 70% in primary islets isolated from mouse pancreas, when compared to 5.5 mM glucose. The suppression by high glucose is partly due to destabilizing Sepp1 mRNA [32]. 

In contrast to the situation in the pancreas, gene expression and secretion of Sepp1 is increased at high glucose concentrations in rat H4IIEC hepatocytes and rat primary hepatocytes [41,42], while treatment with the antidiabetic drug metformin attenuated Sepp1 mRNA expression and secretion, although the applied dose of metformin was far higher than the therapeutic level in humans [41]. On the other hand, insulin can suppress Sepp1 in a dose- and time-dependent manner in cultured hepatocytes [41,42], and Sepp1 mRNA levels were elevated in the liver in fasted (hypoinsulinemic conditions) compared to fed C57BL6J mice [42]. That suggests that the rising secretion of insulin could suppress Sepp1 expression in the liver under normal insulin action. One of the mechanisms is through attenuating the action of peroxisomal proliferator-activated receptor-γ coactivator-1α (PGC-1α) [43], which is the key regulator of hepatic Sepp1 [44]. However, in three mouse diabetes models of insulin action deficiency, including streptozotocin-induced diabetes and liver insulin-receptor knockout, the expression of PGC-1α was strongly induced [45], which could lead to increased Sepp1 expression. Hence, Sepp1 may be overexpressed under diabetic conditions, whether in an insulin deficiency or insulin resistance.

## 4. The Change of Sepp1 Expression with Glucose and Insulin in Models of Targeted Sepp1 Depletion

Using knock-out Sepp1 mice or cells can help in the understanding of the effects of Sepp1 on carbohydrate metabolism. Misu *et al.* studied the role of Sepp1 in the regulation of glucose metabolism and insulin sensitivity in KKAy mice, a model of type 2 diabetes and obesity; knockdown of Sepp1 by siRNA injection resulted in a 30% reduction in Sepp1 protein levels in the liver and blood and improved both glucose intolerance and insulin resistance [42]. *Sepp1^−^*^/−^ mice, the animal model of Sepp1 deletion, created using genomic recombination technology, were viable when maintained on a selenium-sufficient diet. Compared with wild-type C57Bl/6 mice, postprandial plasma levels of insulin were reduced in *Sepp1^−^*^/−^ mice and glucose tolerance and insulin sensitivity were improved, as shown by glucose and insulin loading tests although blood glucose levels remained unchanged. When on an obesity-inducing diet, *Sepp1^−^*^/−^ mice were protected against glucose intolerance and insulin resistance and serum levels of free fatty acids and insulin were significantly reduced [42]. These results hint that Sepp1 might enhance the production and secretion of pancreatic insulin and/or interfere with the action of insulin on its target tissues. Depletion of Sepp1, both *in vitro* and *in vivo*, enhanced insulin-stimulated phosphorylation of Akt and/or the insulin receptor (IR) in hepatocytes, indicating that Sepp1 may be involved in insulin signaling and may disturb glucose metabolism [42]. It should be noted that, as with all essential micronutrients, selenium status appears to have a U-shaped relationship with its health effects [46]. Sepp1 expression in circulation and tissues is very low in gene-knockout animals, while the side effects of selenium on glucose metabolism are more likely related to higher or super-nutritional levels of Sepp1. Hence, the results derived from Sepp1-deletion models cannot be expected to explain the effects of high levels of Sepp1.

Because there is no existing cell culture or animal model in which Sepp1 is overexpressed, Misu *et al.* purified Sepp1 from human plasma, using chromatographic methods, and administered it to mice or cells in culture. Most of the experimental results contrasted with those from Sepp1-deficient mice [42]. For example, in Sepp1-injected mice, endogenous glucose production increased, peripheral glucose disposal decreased, glucose intolerance and insulin resistance were induced and blood insulin levels were significantly elevated. They demonstrated that Sepp1 is positively associated with the development of insulin resistance from both positive and negative sides. They also observed that insulin-stimulated phosphorylation of Akt was reduced in both liver and skeletal muscle in *in vitro* and *in vivo* studies which can impede insulin signaling [42].

Although the above study systematically explored the effect of Sepp1 on insulin resistance and glucose intolerance, notably in hepatocytes and myocytes, several drawbacks impair the ability to explain the relationship of Sepp1 to type 2 diabetes. Firstly, experimental mice were intraperitoneally injected with purified human Sepp1, while controls were injected with PBS, so the stress reaction resulting from species heterology cannot be excluded. Secondly, there is the key question of how Sepp1 enters hepatocytes and myocytes to perform its action. As far as we know, Sepp1 as a kind of glycoprotein that is taken up variably by tissues depending on whether they express the receptors apolipoprotein E receptor 2 (ApoER2) or megalin [47]. For instance, ApoER2 is expressed in testis, bone marrow, placenta, brain, thymus and spleen [48]. In Sertoli cells, ApoER2 mediated endocytosis of Sepp1 and delivered it to lysosomes for digestion to ensure the supply of selenium [49]. Megalin is the receptor for Sepp1 in the kidney; it mediates Sepp1 endocytic uptake by the proximal convoluted tubule cells, preventing its loss in the urine and thereby enabling it to provide selenium for the synthesis of Gpx3 [50]. However, Kurokawa *et al.* [48] reported that ApoER2 mRNA was hardly detectable in mouse liver, and its expression by the quadriceps muscle was only 1.4% that of brain and 0.07% of testis. On the other hand, megalin is an endocytic receptor expressed on the apical surface of several epithelial cells. Erranz *et al*. reported that megalin is present in gallbladder epithelial cells, but not in liver cells [51]. These results raise the question of whether Sepp1 could impair insulin signaling directly on the hepatocyte or myocyte, although they could have an as yet unidentified receptor. Thirdly, in either Sepp1-injected or Sepp1-deletion mice, Misu *et al.* failed to address the effects of the Se-transporter Sepp1 on Se concentration of target tissue and Gpx1 expression, as Gpx1 has already been shown to affect insulin and glucose metabolism [52]. Moreover, they [42] only observed the acute effect (maximum duration is 12 h) of overdose of Sepp1; studies of the long-term effect of overexpressing Sepp1 are required to mimic the situation in human studies. 

## 5. Regulation of Hepatic Sepp1 Expression by Factors Related to Glucose Metabolism

Steinbrenner *et al*. found that Sepp1 in liver is regulated virtually like the gluconeogenic enzymes, glucose-6-phosphatase (G6Pase) and phosphoenolpyruvate carboxykinase (PEP-CK) [44]. The interaction between forkhead box protein class O1a (FoxO1a) and PGC-1α is of crucial importance for transcriptional regulation of the above two enzymes [43,45]; they also stimulate Sepp1 expression in liver [44]. A binding site for the FoxO1a transcription factor located in Sepp1 promoter has been identified; it is in close proximity to a binding site for hepatocyte nuclear factor 4α (HNF-4α) [44,45,53]. High-level transcription of the Sepp1 gene in liver is ensured by the combined action of FoxO1a and HNF-4α with the coactivator (PGC)-1α. Moreover, insulin inhibited Sepp1 transcription via the PI3K/Akt/FoxO1a axis [53], whereas the PGC-1α-inducing glucocorticoid dexamethasone strongly enhanced Sepp1 expression and protein secretion in cultured rat hepatocytes [44]. 

**Figure 1:**
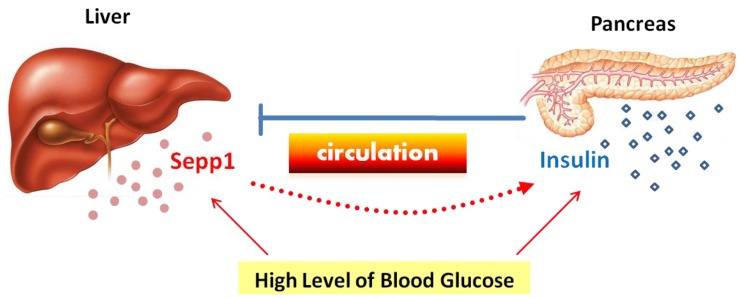
Feedback regulation between hepatic Sepp1 and pancreatic insulin under the normal insulin-sensitive condition. Circulating high glucose concentration stimulates the expression of insulin in the pancreas and Sepp1 in the liver. If the increasing Sepp1 is transported into the pancreas and collaterally enhances insulin production, then sufficient insulin may feedback to inhibit hepatic Sepp1 production.

The evidence suggests that Sepp1 should be negatively regulated by insulin in the liver in the situation of normal insulin sensitivity. We hypothesize that there could be a feedback regulation between hepatic Sepp1 and pancreatic insulin: in the normal organism, circulating high-glucose concentration stimulates the expression of insulin in the pancreas and Sepp1 in the liver. The increasing Sepp1 is transported into the pancreas and may collaterally enhance insulin production, as glucose tolerance tests revealed that blood insulin levels were significantly elevated at 0 min in SEPP1-injected mice in the study of Misu and colleagues [42]. Then sufficient insulin may feedback to inhibit hepatic Sepp1 expression as if it was a gluconeogenic enzyme, to maintain glucose homeostasis (Figure 1). However, if insulin resistance is present, normal insulin signaling would be impaired in hepatocytes leading to failure to suppress Sepp1 expression as well as that of gluconeogenic enzymes. Eventually, Sepp1 and gluconeogenic enzymes will be over-produced and secreted, further increasing the blood glucose level. Hence, we think that the increase in circulating Sepp1 could be the result rather than the cause of abnormal glucose metabolism.

## 6. Sepp1 and Energy Metabolism

Obesity is closely related to insulin resistance and is a strong risk factor in the development of type 2 diabetes. Weight changes have also been observed in several mouse strains with Sepp1-associated gene knockout. In the above *Sepp1^−^*^/−^ mice, normal body weights were maintained on a selenium-sufficient diet, and body weight, food intake, oxygen consumption and lipid accumulation in the liver and adipose tissues were unaffected. When mice were fed a high-fat, high-sucrose diet, Sepp1 knockouts tended to have more body-weight gain, but daily food intake and basal energy expenditure significantly increased and adipocyte hypertrophy attenuated, compared to wild-type mice [42]. *Sepp1^c/c^/alb-cre^+/^*^−^ mice are an animal model of selective deletion of Sepp1 in hepatocytes in which plasma Sepp1 concentration is much lower than in *Sepp1^c/c^* mice (controls). On a selenium-deficient diet, *Sepp1^c/c^/alb-cre^+/^*^−^ mice ceased gaining weight 12 weeks after weaning, although *Sepp1^c/c^* mice kept gaining weight for 22 weeks [20]. 

Recently, a connection between metabolic energy regulation and selenium metabolism through selenocysteinelyase (Scly), which mediates the pathway for recycling selenium, was reported [54]. Scly is the enzyme that supplies selenium for selenoprotein biosynthesis via decomposition of the amino acid Sec [55,56]. Seale *et al.* found that Scly knockout mice on a selenium-adequate diet exhibited hyperinsulinemia, hyperleptinemia, glucose intolerance, and hepatic steatosis, with larger white-adipose-tissue depots, all of which are often associated with the early stages of metabolic syndrome. At the same time, Sepp1 mRNA was obviously upregulated in the liver but the protein level in the circulation remained unchanged. Furthermore, upon dietary selenium restriction, Scly knockout mice developed several characteristics of the metabolic syndrome, such as fatty liver and hypercholesterolemia, with aggravated hyperleptinemia, hyperinsulinemia, and glucose intolerance [54]. Sepp1 mRNA was upregulated but the protein level did not change in the liver, and circulating Sepp1 significantly decreased compared with the control [54]. These studies illustrate that Sepp1 could be involved in energy metabolism, which suggests that the relationship between Sepp1 and glucose metabolism should be explored.

## 7. Conclusions

In summary, Sepp1 is closely related to glucose metabolism, but it is unclear which is the chicken and which is the egg. Based on the present experimental evidence, we speculate that there could be a feedback regulation between hepatic Sepp1 and pancreatic insulin and that increased circulating Sepp1 might be the result rather than the cause of abnormal glucose metabolism. Future studies specifically designed to overexpress Sepp1 are needed in order to provide a more robust link between Sepp1 and type 2 diabetes. However, the sexual dimorphic expression of selenoproteins, including Sepp1, in the various tissues when treated with selenium should be noted and the observed differences appear to change with selenium status and age of the individuals [57]. For example, the increased risk of diabetes was confined to males with very high Se status in NHANES III study [10]. There are currently more questions than answers. Extrapolation from cell culture or mouse studies to humans should be avoided, and even humans need to be separated into well-supplied and selenium-deficient subjects, old and young, males and females.

## References

[1] Schweizer U., Brauer A.U., Kohrle J., Nitsch R., Savaskan N.E. (2004). Selenium and brain function: A poorly recognized liaison. Brain Res. Rev..

[2] Boitani C., Puglisi R. (2008). Selenium, a key element in spermatogenesis and male fertility. Adv. Exp. Med. Biol..

[3] Huang Z., Rose A.H., Hoffmann P.R. (2012). The role of selenium in inflammation and immunity: From molecular mechanisms to therapeutic opportunities. Antioxid. Redox Signal..

[4] Schomburg L. (2012). Selenium, selenoproteins and the thyroid gland: Interactions in health and disease. Nat. Rev. Endocrinol..

[5] Muecke R., Schomburg L., Buentzel J., Kisters K., Micke O. (2010). Selenium or no selenium—That is the question in tumor patients: A new controversy. Integr. Cancer Ther..

[6] Ezaki O. (1990). The insulin-like effects of selenate in rat adipocytes. J. Biol. Chem..

[7] Roden M., Prskavec M., Furnsinn C., Elmadfa I., Konig J., Schneider B., Wagner O., Waldhausl W. (1995). Metabolic effect of sodium selenite: Insulin-like inhibition of glucagon-stimulated glycogenolysis in the isolated perfused rat liver. Hepatology.

[8] Mueller A.S., Pallauf J. (2006). Compendium of the antidiabetic effects of supranutritional selenate doses. *In vivo* and *in vitro* investigations with type ii diabetic *db/db* mice. J. Nutr. Biochem..

[9] Czernichow S., Couthouis A., Bertrais S., Vergnaud A.C., Dauchet L., Galan P., Hercberg S. (2006). Antioxidant supplementation does not affect fasting plasma glucose in the supplementation with antioxidant vitamins and minerals (su.Vi.Max) study in france: Association with dietary intake and plasma concentrations. Am. J. Clin. Nutr..

[10] Bleys J., Navas-Acien A., Guallar E. (2007). Serum selenium and diabetes in U.S. Adults. Diabetes Care.

[11] Laclaustra M., Navas-Acien A., Stranges S., Ordovas J.M., Guallar E. (2009). Serum selenium concentrations and diabetes in U.S. Adults: National health and nutrition examination survey (nhanes) 2003–2004. Environ. Health Perspect..

[12] Stranges S., Galletti F., Farinaro E., D’Elia L., Russo O., Iacone R., Capasso C., Carginale V., de Luca V., Della Valle E. (2011). Associations of selenium status with cardiometabolic risk factors: An 8-year follow-up analysis of the olivetti heart study. Atherosclerosis.

[13] Rajpathak S., Rimm E., Morris J.S., Hu F. (2005). Toenail selenium and cardiovascular disease in men with diabetes. J. Am. College Nutr..

[14] Akbaraly T.N., Arnaud J., Rayman M.P., Hininger-Favier I., Roussel A.M., Berr C., Fontbonne A. (2010). Plasma selenium and risk of dysglycemia in an elderly french population: Results from the prospective epidemiology of vascular ageing study. Nutr. Metabol..

[15] Park K., Rimm E.B., Siscovick D.S., Spiegelman D., Manson J.E., Morris J.S., Hu F.B., Mozaffarian D. (2012). Toenail selenium and incidence of type 2 diabetes in U.S. men and women. Diabetes Care.

[16] Rayman M.P., Blundell-Pound G., Pastor-Barriuso R., Guallar E., Steinbrenner H., Stranges S. (2012). A randomized trial of selenium supplementation and risk of type-2 diabetes, as assessed by plasma adiponectin. PLoS One.

[17] Lippman S.M., Klein E.A., Goodman P.J., Lucia M.S., Thompson I.M., Ford L.G., Parnes H.L., Minasian L.M., Gaziano J.M., Hartline J.A. (2009). Effect of selenium and vitamin E on risk of prostate cancer and other cancers: The selenium and vitamin e cancer prevention trial (select). JAMA.

[18] Klein E.A., Thompson I.M., Tangen C.M., Crowley J.J., Lucia M.S., Goodman P.J., Minasian L.M., Ford L.G., Parnes H.L., Gaziano J.M. (2011). Vitamin E and the risk of prostate cancer: The selenium and vitamin e cancer prevention trial (select). JAMA.

[19] Stranges S., Marshall J.R., Natarajan R., Donahue R.P., Trevisan M., Combs G.F., Cappuccio F.P., Ceriello A., Reid M.E. (2007). Effects of long-term selenium supplementation on the incidence of type 2 diabetes: A randomized trial. Ann. Intern. Med..

[20] Hill K.E., Wu S., Motley A.K., Stevenson T.D., Winfrey V.P., Capecchi M.R., Atkins J.F., Burk R.F. (2012). Production of selenoprotein P (sepp1) by hepatocytes is central to selenium homeostasis. J. Biol. Chem..

[21] Yang S.J., Hwang S.Y., Choi H.Y., Yoo H.J., Seo J.A., Kim S.G., Kim N.H., Baik S.H., Choi D.S., Choi K.M. (2011). Serum selenoprotein P levels in patients with type 2 diabetes and prediabetes: Implications for insulin resistance, inflammation, and atherosclerosis. J. Clin. Endocrinol. Metabol..

[22] Misu H., Ishikura K., Kurita S., Takeshita Y., Ota T., Saito Y., Takahashi K., Kaneko S., Takamura T. (2012). Inverse correlation between serum levels of selenoprotein P and adiponectin in patients with type 2 diabetes. PLoS One.

[23] Saito Y., Sato N., Hirashima M., Takebe G., Nagasawa S., Takahashi K. (2004). Domain structure of bi-functional selenoprotein P. Biochem. J..

[24] Burk R.F., Hill K.E., Motley A.K. (2001). Plasma selenium in specific and non-specific forms. BioFactors.

[25] Akesson B., Bellew T., Burk R.F. (1994). Purification of selenoprotein P from human plasma. Biochim. Biophys. Acta.

[26] Meplan C., Crosley L.K., Nicol F., Beckett G.J., Howie A.F., Hill K.E., Horgan G., Mathers J.C., Arthur J.R., Hesketh J.E. (2007). Genetic polymorphisms in the human selenoprotein P gene determine the response of selenoprotein markers to selenium supplementation in a gender-specific manner (the selgen study). FASEB J..

[27] Xia Y., Hill K.E., Byrne D.W., Xu J., Burk R.F. (2005). Effectiveness of selenium supplements in a low-selenium area of china. Am. J. Clin. Nutr..

[28] Combs G.F., Jackson M.I., Watts J.C., Johnson L.K., Zeng H., Idso J., Schomburg L., Hoeg A., Hoefig C.S., Chiang E.C. (2012). Differential responses to selenomethionine supplementation by sex and genotype in healthy adults. Br. J. Nutr..

[29] Hoffmann P.R., Hoge S.C., Li P.A., Hoffmann F.W., Hashimoto A.C., Berry M.J. (2007). The selenoproteome exhibits widely varying, tissue-specific dependence on selenoprotein P for selenium supply. Nucl. Acids Res..

[30] Kato T., Read R., Rozga J., Burk R.F. (1992). Evidence for intestinal release of absorbed selenium in a form with high hepatic extraction. Am. J. Physiol..

[31] Schweizer U., Streckfuss F., Pelt P., Carlson B.A., Hatfield D.L., Kohrle J., Schomburg L. (2005). Hepatically derived selenoprotein P is a key factor for kidney but not for brain selenium supply. Biochem. J..

[32] Steinbrenner H., Hotze A.L., Speckmann B., Pinto A., Sies H., Schott M., Ehlers M., Scherbaum W.A., Schinner S. (2013). Localization and regulation of pancreatic selenoprotein P. J. Mol. Endocrinol..

[33] Lee S.R., Yon J.M., Baek I.J., Kim M.R., Park C.G., Lee B.J., Yun Y.W., Nam S.Y. (2008). Spatiotemporal expression of the selenoprotein P gene in postimplantational mouse embryos. Int. J. Dev. Biol..

[34] Niwa H., Harrison L.C., DeAizpurua H.J., Cram D.S. (1997). Identification of pancreatic β cell-related genes by representational difference analysis. Endocrinology.

[35] Yang X., Jansson P.A., Nagaev I., Jack M.M., Carvalho E., Sunnerhagen K.S., Cam M.C., Cushman S.W., Smith U. (2004). Evidence of impaired adipogenesis in insulin resistance. Biochem. Biophys. Res. Commun..

[36] Zhang Y., Chen X. (2011). Reducing selenoprotein P expression suppresses adipocyte differentiation as a result of increased preadipocyte inflammation. Am. J. Physiol. Endocrinol. Metab..

[37] Clark L.C., Combs G.F., Turnbull B.W., Slate E.H., Chalker D.K., Chow J., Davis L.S., Glover R.A., Graham G.F., Gross E.G. (1996). Effects of selenium supplementation for cancer prevention in patients with carcinoma of the skin. A randomized controlled trial. Nutritional prevention of cancer study group. JAMA.

[38] Duffield-Lillico A.J., Dalkin B.L., Reid M.E., Turnbull B.W., Slate E.H., Jacobs E.T., Marshall J.R., Clark L.C. (2003). Selenium supplementation, baseline plasma selenium status and incidence of prostate cancer: An analysis of the complete treatment period of the nutritional prevention of cancer trial. BJU Int..

[39] Angstwurm M.W., Engelmann L., Zimmermann T., Lehmann C., Spes C.H., Abel P., Strauss R., Meier-Hellmann A., Insel R., Radke J. (2007). Selenium in Intensive Care (SIC): Results of a prospective randomized, placebo-controlled, multiple-center study in patients with severe systemic inflammatory response syndrome, sepsis, and septic shock. Crit. Care Med..

[40] Campbell S.C., Aldibbiat A., Marriott C.E., Landy C., Ali T., Ferris W.F., Butler C.S., Shaw J.A., Macfarlane W.M. (2008). Selenium stimulates pancreatic beta-cell gene expression and enhances islet function. FEBS Lett..

[41] Speckmann B., Sies H., Steinbrenner H. (2009). Attenuation of hepatic expression and secretion of selenoprotein P by metformin. Biochem. Biophys. Res. Commun..

[42] Misu H., Takamura T., Takayama H., Hayashi H., Matsuzawa-Nagata N., Kurita S., Ishikura K., Ando H., Takeshita Y., Ota T. (2010). A liver-derived secretory protein, selenoprotein P, causes insulin resistance. Cell Metab..

[43] Puigserver P., Rhee J., Donovan J., Walkey C.J., Yoon J.C., Oriente F., Kitamura Y., Altomonte J., Dong H., Accili D. (2003). Insulin-regulated hepatic gluconeogenesis through foxo1-pgc-1α interaction. Nature.

[44] Speckmann B., Walter P.L., Alili L., Reinehr R., Sies H., Klotz L.O., Steinbrenner H. (2008). Selenoprotein P expression is controlled through interaction of the coactivator pgc-1α with foxo1a and hepatocyte nuclear factor 4α transcription factors. Hepatology.

[45] Yoon J.C., Puigserver P., Chen G., Donovan J., Wu Z., Rhee J., Adelmant G., Stafford J., Kahn C.R., Granner D.K. (2001). Control of hepatic gluconeogenesis through the transcriptional coactivator pgc-1. Nature.

[46] Rayman M.P. (2012). Selenium and human health. Lancet.

[47] Burk R.F., Hill K.E. (2009). Selenoprotein P—expression, functions, and roles in mammals. Biochim. Biophys. Acta.

[48] Kurokawa S., Hill K.E., McDonald W.H., Burk R.F. (2012). Long isoform mouse selenoprotein P (sepp1) supplies rat myoblast l8 cells with selenium via endocytosis mediated by heparin binding properties and apolipoprotein e receptor-2 (apoer2). J. Biol. Chem..

[49] Olson G.E., Winfrey V.P., Nagdas S.K., Hill K.E., Burk R.F. (2007). Apolipoprotein e receptor-2 (apoer2) mediates selenium uptake from selenoprotein P by the mouse testis. J. Biol. Chem..

[50] Olson G.E., Winfrey V.P., Hill K.E., Burk R.F. (2008). Megalin mediates selenoprotein P uptake by kidney proximal tubule epithelial cells. J. Biol. Chem..

[51] Erranz B., Miquel J.F., Argraves W.S., Barth J.L., Pimentel F., Marzolo M.P. (2004). Megalin and cubilin expression in gallbladder epithelium and regulation by bile acids. J. Lipid Res..

[52] Lei X.G., Vatamaniuk M.Z. (2011). Two tales of antioxidant enzymes on beta cells and diabetes. Antioxid. Redox Signal..

[53] Walter P.L., Steinbrenner H., Barthel A., Klotz L.O. (2008). Stimulation of selenoprotein P promoter activity in hepatoma cells by foxo1a transcription factor. Biochem. Biophys. Res. Commun..

[54] Seale L.A., Hashimoto A.C., Kurokawa S., Gilman C.L., Seyedali A., Bellinger F.P., Raman A.V., Berry M.J. (2012). Disruption of the selenocysteine lyase-mediated selenium recycling pathway leads to metabolic syndrome in mice. Mol. Cell. Biol..

[55] Collins R., Johansson A.L., Karlberg T., Markova N., van den Berg S., Olesen K., Hammarstrom M., Flores A., Schuler H., Schiavone L.H. (2012). Biochemical discrimination between selenium and sulfur 1: A single residue provides selenium specificity to human selenocysteine lyase. PLoS One.

[56] Mihara H., Kurihara T., Watanabe T., Yoshimura T., Esaki N. (2000). Cdna cloning, purification, and characterization of mouse liver selenocysteine lyase. Candidate for selenium delivery protein in selenoprotein synthesis. J. Biol. Chem..

[57] Schomburg L., Schweizer U. (2009). Hierarchical regulation of selenoprotein expression and sex-specific effects of selenium. Biochim. Biophys. Acta.

